# Variations in Opioid Prescribing Behavior by Physician Training

**DOI:** 10.5811/westjem.2019.3.39311

**Published:** 2019-04-16

**Authors:** Evan L. Leventhal, Larry A. Nathanson, Alden M. Landry

**Affiliations:** Beth Israel Deaconess Medical Center, Harvard Medical School, Department of Emergency Medicine, Boston, Massachusetts

## Abstract

**Introduction:**

Opioid abuse has reached epidemic proportions in the United States. Patients often present to the emergency department (ED) with painful conditions seeking analgesic relief. While there is known variability in the prescribing behaviors of emergency physicians, it is unknown if there are differences in these behaviors based on training level or by resident specialty.

**Methods:**

This is a retrospective chart review of ED visits from a single, tertiary-care academic hospital over a single academic year (2014–2015), examining the amount of opioid pain medication prescribed. We compared morphine milligram equivalents (MME) between provider specialty and level of training (emergency medicine [EM] attending physicians, EM residents in training, and non-EM residents in training).

**Results:**

We reviewed 55,999 total ED visits, of which 4,431 (7.9%) resulted in discharge with a prescription opioid medication. Residents in a non-EM training program prescribed higher amounts of opioid medication (108 MME, interquartile ratio [IQR] 75–150) than EM attendings (90 MME, lQR 75–120), who prescribed more than residents in an EM training program (75 MME, IQR 60–113) (p<0.01).

**Conclusion:**

In an ED setting, variability exists in prescribing patterns with non-EM residents prescribing larger amounts of opioids in the acute setting. EM attendings should closely monitor for both over- and under-prescribing of analgesic medications.

## INTRODUCTION

Harm from prescription opioid misuse and overdose has increased to epidemic proportions in the United States.^1^,^2^ Emergency physicians (EP) are often perceived to over-prescribe opioid analgesic medications,^3^ thus contributing to the current public health crisis. Patients often turn to the emergency department (ED) for treatment of a variety of painful conditions, many of whom are discharged with analgesic prescriptions.^4^,^5^

Wide variations between specialties exist in prescribing patterns.^6^ Acute pain is a typical cause of ED visits, leading EPs to commonly prescribe opioid prescriptions.^5^ However, most of these prescriptions from the ED have a comparatively low pill count and a relatively small total amount of opioids.^7^ Despite this, we now know there is no known safe dosing, and addiction can occur even after a short course of treatment.^8^,^9^ While there have been many initiatives to provide alternative treatments to treat severe pain, opioids continue to have their place in providing appropriate analgesia.^10^ There is known variability in the opioid prescribing patterns of EPs.^11^

It is not known if there is a difference in opioid prescribing patterns from the ED between providers of different training levels and specialty training. This study aimed to determine whether variations in physician characteristics correlated with increased amounts of opioid prescription quantities. We hypothesized that providers with the most training and experience in the ED setting – emergency medicine (EM) attendings – would prescribe the smallest amount of opioids. Conversely, we hypothesized that residents in non-EM training programs would be less likely to be familiar with ED practices and populations, and would therefore prescribe the largest quantity of opioids.

## METHODS

### Study Design and Setting

This was a retrospective chart review of all patients discharged from a single, urban, academic Level 1 trauma center and tertiary referral center with approximately 56,000 annual visits. All patients seen during a single academic year (June 1, 2014–June 30, 2015) were included in the study. Because it is an academic medical center, most patients are seen by a resident physician who is training either in EM or another specialty. All patients are seen by a supervising attending physician who is board certified in EM. Physician assistants and nurse practitioners did not see ED patients at this site during the study time period. This study was approved by the local institutional review board.

### Patient Selection

We reviewed the charts of all patients discharged from the ED during the study period, and those with a prescription for opioid pain medications were included in this study. We excluded prescriptions for transdermal opioid medication (eg, fentanyl patches). Prescriptions with missing or invalid information were also excluded from the study.

### Methods and Measurements

For each visit, we digitally extracted the following data from the electronic medical record (EMR): patient age, gender, triage Emergency Severity Index (ESI), chief complaint, pain score at time of triage (0 to 10), and any prescription for an opioid pain medication at the time of discharge. We classified combination medications (eg, those containing acetaminophen as well as an opioid) by their opioid ingredient alone. The ingredients of each medication were determined from the First Databank Drug Database (First Databank, South San Francisco, California). The quantity of prescribed opioid was reported in morphine milligram equivalents (MME) (median and interquartile ratio [IQR]) using standard conversion tables.^12^ Chief complaints with sidedness specified had their sidedness removed (eg, “left wrist pain” was changed to “wrist pain”), but were otherwise grouped together unchanged.

The discharge module of our EMR allows for discharge planning to be started and modified throughout the patient visit. All discharge prescriptions are entered electronically, and are then printed on paper at the time of discharge. The EMR records the prescribing provider who entered the prescription plan into the EMR. For study purposes, this “prescribing provider” was identified and classified as either part of the primary team who first saw the patient and initiated the workup, or as part of a sign-out team who took over care for the patient at change of shift and who typically would complete a pre-established plan of care.

We grouped prescribing providers based on medical specialty and level of training: EM attending physicians, EM residents, and non-EM residents. During the study period, all providers had the ability to independently prescribe opioids. There was no opioid prescribing policy that constrained either the type or quantity of opioids prescribed. All of the relevant data fields were fully codified allowing for the extraction and classification to be automated.

**Population Health Research Capsule**

What do we already know about this issue?

*Opioid abuse represents an important health crisis in the United States. There are known variations in provider prescribing behavior from the emergency department*.

What was the research question?

Do physicians of different training level and resident specialty prescribe different quantities of opioids?

What was the major finding of the study?

*Non-emergency medicine residents prescribed more opioids than emergency-medicine trained providers*.

How does this improve population health?

*By better understanding inherent prescribing trends, we can better inform those attempting to characterize and modify current practice*.

Our EMR does not contain defaults for prescription quantities. Instead, it maintains a dynamic list of the most frequently written prescriptions, which are provided as shortcuts. The EMR does provide a list of suggested prescriptions grouped by condition. The only condition in our EMR that had any opioid listed during the study period was “back pain,” which listed oxycodone-acetaminophen (5 milligrams (mg-325 mg) dispense 10 tablets (75 MME) as well as hydrocodone-acetaminophen (5–325 mg) dispense 15 tablets (75 MME).

### Data Analysis

The primary outcome measure was the difference in prescribing quantities between the three groups of providers. We tested differences in these quantities using a one-way analysis of variance,, and we made pairwise comparisons using the posthoc Tukey test. Statistical analysis was performed with Python 3.6.3, using the open-source Pandas and SciPy library of packages.^13^–^15^ A p value of <0.05 was considered statistically significant. We used descriptive statistics to look at the specific opioid drug prescribed.

## RESULTS

We reviewed a total of 55,999 ED patient visits, and of those, 32,968 resulted in a discharge for outpatient care. Of those discharged, 4,431 visits included a prescription for an opioid medication (8% of all visits and 13% of discharged patient visits). No patient received more than one opioid prescription at the time of discharge. Two prescriptions for transdermal patches were excluded from the study. We also excluded 17 prescriptions that could not be filled due to invalid data elements: two were for controlled-substance refills, which is not permissible, while 15 of the excluded prescriptions specified invalid or nonspecific dispensing quantities.

**Figure 1:**
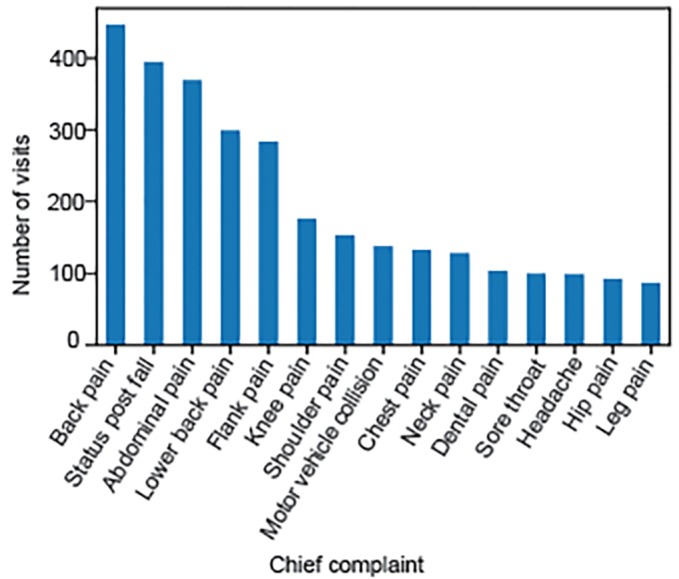
Most common chief complaints. Back pain was the most common chief complaint for which an opioid prescription was written. Combined, the top 15 chief complaints accounted for 52% of the opioid prescriptions.

The median age of included patients was 45 (interquartile ratio [IQR] 32–58). The median triage ESI was 3 (IQR 3–3), and the median pain score at the time of triage was 8 (IQR 6–10). More complete demographics for the study participants are included in Table 1. The most common chief complaints, which resulted in an opioid prescription at discharge, were “back pain,” followed by “fall” and “abdominal pain” (Figure 1).

**Figure 2:**
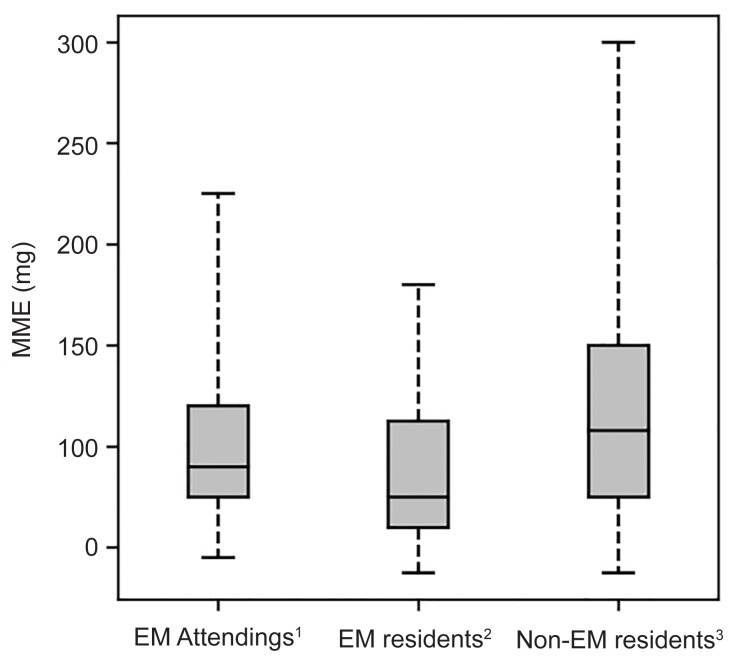
Total morphine milligram equivalent per patient by prescriber group. Quantity of opioids prescribed in morphine milligram equivalent (MME) by each group of providers. Gray box shows the median and interquartile range, while the whisker lines represent the 95th percentile. *EM*, emergency medicine; *mg*, milligram.

There were significant differences in the amount of opioid pain medication prescribed between the three groups (p<0.01) (Figure 2). EM residents prescribed the least amount (75 MME, IQR 60–113), while non-EM residents prescribed the largest amount of opioid analgesic (108 MME, IQR 75–150). EM attendings (90 MME, lQR 75–120) prescribed less than the non-EM resident providers and more than EM residents. Less than 1% of prescriptions were for extended-release formulations, and 83% of the prescriptions were for oxycodone (Table 2).

There were three outlier patients in the study. Two patients were prescribed high quantities of opioids by EM attendings for palliative care (5800 and 1900 MME). One patient was prescribed 1400 MME by a non-EM resident for postoperative pain at the request of consulting service. Inclusion of these three patients had no statistical effect.

## DISCUSSION

Our study showed that physician specialty and level of training influence the amount of opioids prescribed from the ED. EPs, both residents and attendings, prescribe smaller quantities of opioid analgesic medications than non-EM trained providers in the ED. In contrast to our original hypothesis, EM residents prescribed fewer opioid quantities than EM attendings.

It is not clear why EM residents prescribed fewer opioids than the EM attendings. One possibility is that many of the attendings trained and practiced during the time when there was a greater push towards treating discomfort, and accreditation organizations were emphasizing pain evaluation and reduction.^16^ By contrast, most residents have only practiced during the current opioid crisis and may be more ingrained with the concept of minimizing opioid prescribing. Overall, the total amount of opioids prescribed was quite low, consistent with prior research. Immediate-release oxycodone was overwhelmingly the primary medication prescribed; very few extended-release medications were prescribed. This is consistent with known prescribing behaviors of EPs.^7^

The dynamic nature of our frequently used prescriptions makes it difficult to measure the effect this bias would have on prescribing behaviors. However, as all providers see the same dynamic list, one would expect this to bring prescribing patterns closer together for all providers and be likely to bias the results toward the null hypothesis. While there was a statistically significant difference in the amount of opioids prescribed between each group, the overall prescribing quantities were generally small. Further, the difference in median MME prescribed between the highest and lowest groups was 33 MME, which converts to only four and a half tablets of the most common – oxycodone 5 mg tablets. While there is no safe dose, it is unclear whether such a small difference is clinically significant. At this time, the optimal amount of opioids to prescribe is unknown: too much and we risk contributing to the opioid epidemic,^1^ and if too little we risk undertreating patients in acute pain.

## LIMITATIONS

Our study was limited to a single, tertiary, academic medical center and its generalizability to other institutions must be carefully considered. As a retrospective analysis, unmeasured confounders may have biased the analysis. Only the final discharge prescription was evaluated in this study. Additionally, opioid amounts were attributed solely to the provider who entered the final prescription into the EMR. This means we were unable to measure the effect of any discussion held between the resident trainee and the attending, nor were we able to measure a change in the planned prescribing amount prior to the patient’s discharge. So, while this may not reflect what a resident had independently planned on prescribing, it accurately reflects the final supervised care.

## CONCLUSION

We noted small but statistically significant variations in opioid prescribing practice between providers of different levels of training and specialty. While we did not explore the rationale for prescribing doses, we did note that those who are or have been trained in EM tend to prescribe lower doses of opiate therapy. As always, EM attendings must be cognizant of a trainee’s opioid prescribing patterns.

## Figures and Tables

**Table 1:** Characterisitics of patients discharged with opioid prescriptions.

	All (n=4425)	EM Attendings^1^ (n=527)	EM residents^2^ (n=3089)	Non-EM residents^3^ (n=809)
Age (median [IQR])	45 [32–58]	44 [30–58]	46 [32–58]	46 [34–58]
Gender				
% female (n)	56% (2495)	53% (277)	56% (1742)	59% (476)
% male (n)	44% (1930)	47% (250)	44% (1347)	41% (333)
ESI				
1 % (n)	3.1% (138)	1.5% (8)	4.0% (123)	0.9% (7)
2 % (n)	14.2% (627)	9.9% (52)	15.3% (473)	12.6% (102)
3 % (n)	69.6% (3078)	68.7% (362)	68.0% (2102)	75.9% (614)
4 % (n)	12.9% (569)	18.6% (98)	12.5% (386)	10.5% (85)
5 % (n)	0.3% (13)	1.3% (7)	0.2% (5)	0.1% (1)
First pain score (median [IQR])	8 [6–10]	8 [6–9]	8 [6–10]	8 [7–10]
Prescribed by primary team % (n)	86% (3787)	80% (419)	86% (2663)	87% (705)

*EM*, emergency medicine; *IQR*, interquartile range; *ESI*, Emergency Severity Index.

**Table 2:** Distribution of prescriptions based on their opioid ingredient.

	All (n=4425)	EM Attendings^1^ (n=527)	EM residents^2^ (n=3089)	Non-EM residents^3^ (n=809)
Codeine % (N)	1% (52)	2% (9)	1% (31)	1% (12)
Hydrocodone % (N)	6% (263)	8% (40)	6% (179)	5% (44)
Hydromorphone % (N)	3% (147)	3% (14)	3% (101)	4% (32)
Methadone % (N)	<1% (1)	0% (0)	<1% (1)	0% (0)
Morphine % (N)	<1% (9)	1% (3)	0% (0)	1% (6)
Morphine ER % (N)	<1% (5)	0% (0)	<1% (2)	<1% (3)
Oxycodone % (N)	83% (3685)	82% (434)	85% (2614)	79% (637)
Oxycodone ER % (N)	<1% (4)	0% (0)	<1% (1)	<1% (3)
Tramadol % (N)	6% (259)	5% (27)	5% (160)	9% (72)

*EM*, emergency medicine; *ER*, extended release.
